# Overweight, Obesity, Hypertriglyceridemia, and Insulin Resistance Are Positively Associated with High Serum Copper Levels in Mexican Adults

**DOI:** 10.3390/metabo14050282

**Published:** 2024-05-14

**Authors:** Armando Ramírez-Cruz, María Judith Rios-Lugo, Jacqueline Soto-Sánchez, Cuauhtémoc Arturo Juárez-Pérez, Alejandro Cabello-López, Carmina Jiménez-Ramírez, Consuelo Chang-Rueda, Miguel Cruz, Héctor Hernández-Mendoza, Miguel Vazquez-Moreno

**Affiliations:** 1 Unidad de Investigación Médica en Bioquímica, Hospital de Especialidades, Centro Médico Nacional Siglo XXI, Instituto Mexicano del Seguro Social, Ciudad de México 06720, Mexico; ramirez@xanum.uam.mx (A.R.-C.);; 2 Posgrado en Biología Experimental, Universidad Autónoma Metropolitana, Unidad Iztapalapa, Ciudad de México 09340, Mexico; 3 Facultad de Enfermería y Nutrición, Universidad Autónoma de San Luis Potosí, San Luis Potosí 78290, Mexico; judith.rios@uaslp.mx; 4 Sección de Medicina Molecular y Traslacional, Centro de Investigación en Ciencias de Salud y Biomedicina, Universidad Autónoma de San Luis Potosí, San Luis Potosí 78210, Mexico; 5 Sección de Estudios de Posgrado e Investigación, Escuela Nacional de Medicina y Homeopatía, Instituto Politécnico Nacional, Ciudad de México 07320, Mexico; 6 Unidad de Investigación de Salud en el Trabajo, Centro Médico Nacional Siglo XXI, Instituto Mexicano del Seguro Social, Ciudad de México 06720, Mexico; 7 Unidades Médicas de Alta Especialidad, Dr. Victorio de la Fuente Narváez, Instituto Mexicano del Seguro Social, Ciudad de México 07760, Mexico; 8 Facultad de Ciencias Químicas, Campus IV, Universidad Autónoma de Chiapas, Tapachula 30792, Mexico; 9 Instituto de Investigación de Zonas Desérticas, Universidad Autónoma de San Luis Potosí, Altair 200, San Luís Potosí 78377, Mexico; 10 Laboratorio del Agua y Monitoreo Ambiental, Universidad del Centro de México, San Luis Potosí 78250, Mexico

**Keywords:** copper, overweight, obesity, hypertriglyceridemia, insulin resistance, Mexico

## Abstract

Recently, the role of trace elements in the pathophysiology of obesity, insulin resistance (IR), and metabolic diseases has been explored. In this cross-sectional study, we aimed to assess the association of overweight, obesity, and cardiometabolic traits with serum copper (Cu) levels in 346 Mexican adults. Serum Cu level was measured by inductively coupled plasma mass spectrometry (ICP-MS). Anthropometrical data were collected, and biochemical parameters were measured. The triglyceride-glucose (TyG) index was used as a surrogate marker to evaluate IR. Overweight and obesity status was positively associated with the serum Cu level (β = 19.434 ± 7.309, *p* = 0.008). Serum Cu level was observed to have a positive association with serum triglycerides level (β = 0.160 ± 0.045, *p* < 0.001) and TyG (β = 0.001 ± 0.001, *p* < 0.001). Additionally, high serum Cu level was positively associated with overweight and obesity status (odds ratio [OR] = 1.9, 95% confidence interval [95% CI] 1.1–3.4, *p* = 0.014), hypertriglyceridemia (OR = 3.0, 95% CI 1.7–5.3, *p* < 0.001), and IR (OR = 2.6, 95% CI 1.4–4.6, *p* = 0.001). In conclusion, our results suggest that overweight, obesity, hypertriglyceridemia, and IR are positively associated with serum Cu levels in Mexican adults.

## 1. Introduction

Regardless of ethnicity, socioeconomic status, age, and sex, the prevalence of overweight and obesity has increased worldwide [1]. It is even estimated that if these trends continue, half of the global population may be individuals that are overweight or obesity by 2035 [2]. In Mexico, the last National Survey of Health and Nutrition reported a combined prevalence of overweight and obesity of 75.2% in the adult population [3]. Due to its high association with the development of metabolic diseases (insulin resistance [IR], type 2 diabetes, and cardiovascular diseases), the study of risk factors related to overweight and obesity is considered of high importance to improve its prevention and treatment [4].

Recently, the role of trace elements such as iron, manganese, selenium, iodine, zinc, chromium, copper (Cu), iron, and boron in the pathophysiology of obesity, IR, and metabolic diseases has been explored [5,6]. These reports indicate that the deficiency or overload of these trace elements may negatively affect systemic homeostasis related to energy metabolism [7]. In this way, high serum Cu levels have been associated with abnormal glucose homeostasis in the U.S. adult population [5] and positively associated with overweight and obesity in Mexican children [8].

Although it is known that Cu plays an important role in the human antioxidant system because it is a central component of superoxide dismutase, an antioxidant enzyme [9], its implications in the physiological dysregulation related to metabolic diseases are not entirely clear. The deficient concentration of Cu is associated with impaired ATP synthesis and decreased insulin secretion in pancreatic β-cells [10,11]. On the other hand, excessive levels have been associated with an increase in reactive oxygen species, leading to oxidative stress and the progression of IR and diabetes [10,12].

IR is physiologically defined as the inability of target tissues to respond to the action of insulin and is the pathogenic trigger for type 2 diabetes, non-alcoholic fatty liver disease, and atherosclerosis [13]. In recent years, IR diagnosis has increased to evaluate the risk of developing more serious complications related to overweight and obesity. However, because the reference method for quantifying IR is invasive, and the most commonly used alternative methods may be expensive, the determination of IR does not frequently occur in clinical practice at the first level of care of developing populations. Although the hyperinsulinaemic euglycemic clamp has been considered as the reference method to determine IR, it requires the infusing of insulin and dextrose to evaluate correct glucose metabolism [14]. On the other hand, the commonly used methods, the homeostasis model assessment of insulin resistance (HOMA-IR) and the quantitative insulin sensitivity check index (QUICKI), may be less invasive than the reference method. However, these methods involve the measurement of serum insulin, which for some populations in developing countries (people without health insurance and/or with low income), could still represent a high cost and effort to obtain an IR diagnosis [15]. For this reason, in the last years, different methods have been proposed as surrogate markers of IR [15]. One of these is the triglycerides/glucose index, which comes from the arithmetic calculation: Ln (fasting triglycerides (mg/dL) × fasting blood glucose (mg/dL)/2) [16]. The rationale for using the TyG index is that high levels of serum glucose and triglycerides are highly related to elevated levels of circulating free fatty acids, chronic low-grade inflammation, and oxidative stress, which together are characteristic conditions of the pathophysiology of IR [17,18,19]. The risk of IR measured by the TyG index has been used to evaluate harmful long-term cardiovascular events in recent follow-up studies of large cohorts [20].

The obesity pathophysiology implies a state of oxidative stress and chronic low-grade inflammation that is highly related to the dysfunction of insulin signaling and lipid metabolism [21]. In this way, the Mexican adult population could be considered at high risk of developing type 2 diabetes and dyslipidemia due to their high prevalence of overweight and obesity. Furthermore, although its physiological mechanism is not yet fully described, the association between Cu and metabolic health is acknowledged. For this reason, our study hypothesizes that being overweight and obesity or having any cardiometabolic traits will be related to high serum Cu levels. Hence, the present study aimed to analyze the association of overweight and obesity status and cardiometabolic traits (serum levels of fasting plasma glucose [FPG], TC, TG, and TyG index) with serum Cu levels in a sample of Mexican adults.

## 2. Materials and Methods

### 2.1. Study Sample

A total of 346 Mexican adults (214 women and 132 men) between 22 and 57 years from 4 Provinces (San Luis Potosi, Tlaxcala, Chiapas, and Mexico City) were randomly selected and invited to participate in this cross-sectional study between 2018 and 2020. This study population was composed of university students, university professors, and potters. Exclusion criteria for the study included men and women over 60 years of age, subjects with renal disease, hypo- and hyperthyroidism, cancer, hypertension, liver disease, and those individuals with FPG concentrations higher than 126 mg/dL.

### 2.2. Ethical Approval

The data analyzed in the present study are part of protocols that were conducted according to the guidelines established in the Declaration of Helsinki and based on the Regulations of the General Health Law on Health Research in Mexico, based on articles 14 and 16. The Ethics Committees that approved the research protocols were: Bioethics Committee of the National School of Biological Sciences, IPN (Approval register: ENCB/CEI/064/202); Institutional Review Board of the Tapachula School Medicine of UNACH (Approval register: 03/MHT/RPR/087/17); Ethics Committee of the Faculty of Nursing and Nutrition, UASLP (Approval register: 2014–092); and Ethics Committee of the Mexican Social Security Institute (Approval register: CONBIOETICA-09-CEI-009-20160601). In addition, all subjects were asked to approve their written consent. 

### 2.3. Anthropometric and Biochemicals Measurements

Weight and height were measured using a digital scale (Seca, Hamburg, Germany) and a portable stadiometer (Seca 225, Hamburg, Germany). Body mass index (BMI) was calculated as weight (kg)/height (m)^2^. 

After 8 to 12 h of fasting, blood samples were collected from each participant. Subsequently, the blood samples were centrifugated (1600× *g* for 10 min) to obtain serum. Finally, the serum was stored at −80 °C until analysis of Cu levels and cardiometabolic traits. To determine FPG, TG, and TC, a clinical chemistry analyzer (ILAB 300, Instrumentation Laboratory, Barcelona, Spain) was used. TyG index was calculated and used as a surrogate quantification of IR [16,22].

### 2.4. Definition of Binary Cardiometabolic Traits

According to the criteria put forth by the World Health Organization, normal weight, overweight, and obesity status were considered as having BMI < 25 kg/m^2^, ≥ 25 BMI < 30 kg/m^2^, and BMI ≥ 30 kg/m^2^, respectively. To obtain a better visualization of the effect of body weight on serum Cu levels, we proposed an overweight + obesity (OW + OB) variable by arithmetically adding the frequency of overweight individuals plus the frequency of individuals with obesity. According to the reference values suggested in previous reports in the literature, hyperglycemia, hypercholesterolemia, and hypertriglyceridemia were considered as having FPG ≥ 100 mg/dL, TC ≥ 200 mg/dL, and TG ≥ 150 mg/dL, respectively [23,24,25]. Due to the lack of consensus regarding the reference value for IR measured by the TyG index in Mexicans, for this study, we created groups of low, medium, and high TyG index (according to the tertiles of the row data: Low ≤ 8.5; Medium = 8.5–8.9; High ≥ 8.9). We considered individuals with IR as those who presented the highest values of the TyG index in the study, which corresponds to the third tertile of TyG index values (TyG ≥ 8.9).

### 2.5. Serum Cu Quantification by ICP-MS

Serum Cu level quantification was carried out with Inductively Coupled Plasma Mass Spectrometry (ICP-MS iCAP Q, Thermo Scientific, Bremen, Germany) in the kinetic energy discrimination (KED) mode. The amount of volume analyzed was 200 μL of serum following the protocol proposed by Rios-Lugo et al. [22]. The destruction of the organic matter in the sample was performed using acid digestion in a microwave system (MARS6 CEM, Matthews, NC, USA). All samples were recovered and diluted to 10 mL with 2% *v*/*v* high-purity HNO_3_ for Cu analysis by ICP-MS. Finally, Cu quantification was realized by an external calibration curve of Cu (10, 25, 50, 100, 150, 200, 500, and 1000 μg L^−1^). For total serum, the Cu concentration obtained considered the final volume, serum volume, blanks of samples, and recovery of an internal standard of indium (1 μg L^−1^). Concentrated high-purity nitric acid (Milestone Duopur system Milestonesrl, Sorisole, Italia) and high-purity water with >18 MΩ cm (Milli-Q^®^ system Millipore, Mexico City, Mexico) were used in all processes. The Cu standard used in this study was obtained from the High-Purity Standards.

### 2.6. Statically Analysis

The Shapiro–Wilk test was used to evaluate normality in the continuous data [26] and the rank-based inverse normal transformation was used to transform the variables without normal distribution (BMI, serum levels of FPG, TC, TG, Cu, and TyG index; Appendix A). Student’s t- and Chi-square tests were used to compare continuous data and frequencies between individuals with normal weight and individuals that were overweight and obese. To evaluate the association of overweight and obesity status with serum Cu levels, a linear regression model adjusted for age, sex, and locality was employed. The association of cardiometabolic traits as continue variable (BMI, FPG, TC, TG, and TyG index) with serum Cu level was evaluated with linear regression in two models. Model 1: adjusted for age, sex, and locality. Model 2: adjusted for age, sex, locality, and overweight and obesity status. A logistic regression model adjusted for age, sex, locality, overweight and obesity status, was used to evaluate the association of binary cardiometabolic traits (overweight and obesity status, hyperglycemia, hypercholesterolemia, hypertriglyceridemia, and IR) with high serum Cu levels. The association analysis of overweight and obesity status with high serum Cu levels only was adjusted for age, sex, and locality. Based on the raw data, we created tertiles of serum Cu (Tertile 1 ≤ 69.418 µg dL^−1^; Tertile 2 = 69.418–129.397 µg dL^−1^; Tertile 3 ≥ 129.397 µg dL^−1^). Individuals in the tertile 3 (≥129.397 µg dL^−1^) were considered with high serum Cu levels. The software SPSS (version 22.0, IBM, Armonk, NY, USA) was used to perform the statistical analysis and a two-sided *p*-value < 0.05 was considered significant.

## 3. Results

### 3.1. Description of the Study Samples

Table 1 shows the general characteristics of 346 Mexican adults, 107 (30.9%) with normal weight and 239 (69.1%) with overweight and obesity. The variables, BMI, FPG, TC, TG, and TyG index were significantly higher in the overweight and obesity group with respect to the normal weight group (*p* ≤ 0.007). The frequency of women and age were no different between groups (*p* ≥ 0.264). 

### 3.2. Association of Overweight and Obesity Status with Serum Cu Level

**Figure 1:**
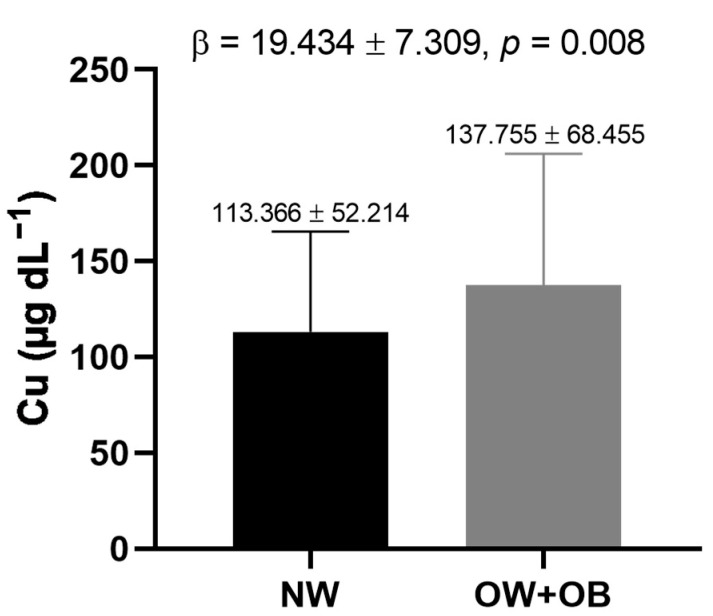
Association of overweight and obesity with serum Cu levels. Analysis by linear regression model adjusted for age, sex, and location. Abbreviations: Cu, copper; NW, normal weight; OW + OB, overweight and obesity status. Sample size: NW = 107; OW + OB = 239.

The mean serum Cu level was higher in subjects with overweight and obesity (137.755 ± 68.455 μg dL^−1^) compared with the normal weight group (113.366 ± 52.214 μg dL^−1^; *p* = 0.001; Figure 1). In a linear regression model adjusted for age, sex, and locality, overweight and obesity status were positively associated with serum Cu levels (β = 19.434 ± 7.309, *p* = 0.008, Figure 1).

### 3.3. Association between Serum Cu Level and Cardiometabolic Traits

The association analysis between cardiometabolic traits and serum Cu levels, before and after adjustment for overweight and obesity status, is presented in Table 2. No significant association was found between BMI, FPG, TC, and serum Cu levels, before (*p* ≥ 0.055) and after (*p* ≥ 0.175) adjustment for overweight and obesity status. The TG level and TyG index were significantly associated with increased serum Cu level (TG: β = 0.188 ± 0.045, *p* < 0.001; TyG: β = 0.002 ± 0.001, *p* < 0.001). The positive association of the TG level and TyG index with serum Cu level remained significant after adjustment for overweight and obesity status (TG: β = 0.160 ± 0.045, *p* < 0.001; TyG: β = 0.001 ± 0.001, *p* < 0.001).

### 3.4. Association of Binary Cardiometabolic Traits with High Serum Cu Level

**Figure 2:**
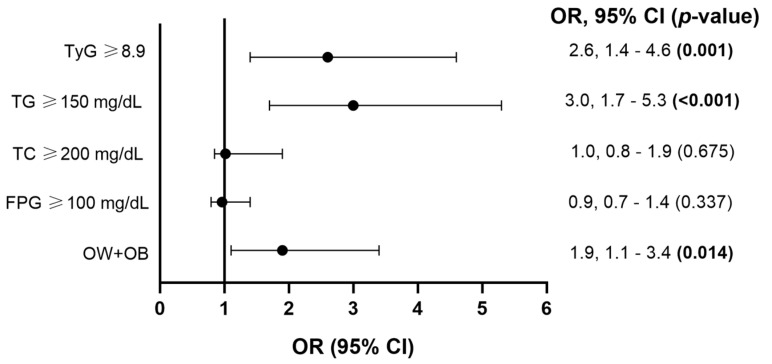
Association of binary cardiometabolic traits (overweight and obesity status [OW + OB], hyperglycemia [FPG ≥ 100 mg/dL], hypercholesterolemia [TC ≥ 200 mg/dL], hypertriglyceridemia [TG ≥ 150 mg/dL], and IR [TyG ≥ 8.9]) with high serum Cu level. Abbreviations: FPG, fasting plasma glucose; TC, total cholesterol; TG, triglycerides; TyG, triglycerides glucose index; OR, odds ratio, 95% CI, 95% confidence interval. Analysis by logistic regression model adjusted for age, sex, locality, and overweight and obesity status. Significant *p*-values (<0.05) are represented in bold. The association analysis of overweight and obesity status with high serum Cu level only was adjusted for age, sex, and locality.

We assessed the association of binary cardiometabolic traits (overweight and obesity status, hyperglycemia, hypercholesterolemia, hypertriglyceridemia, and IR) with high serum Cu level (Figure 2). No significant association was found between hyperglycemia (*p* = 0.337), hypercholesterolemia (*p* = 0.675), and high serum Cu level (Figure 2). However, overweight and obesity status (odds ratio [OR] = 1.9, 95% confidence interval [95% CI] 1.1–3.4, *p* = 0.014), hypertriglyceridemia (OR = 3.0, 95% CI 1.7–5.3, *p* <0.001), and IR (OR = 2.6, 95% CI 1.4–4.6, *p* = 0.001) were positively associated with high serum Cu levels (Figure 2).

## 4. Discussion

In the present study, we evaluated the association of overweight and obesity status and cardiometabolic traits with serum Cu levels. Our results suggest that overweight and obesity status, hypertriglyceridemia, and IR are positively associated with high serum Cu levels.

Cu is a trace element with an important role in antioxidant action and cellular metabolism, and according to balance studies, a requirement of 1.3 mg/day has been suggested for the adult population [27]. In this way, while a deficiency in Cu is associated with anemia and leukopenia, high Cu is associated with liver damage, abdominal pain, cramps, nausea, and diarrhea [27,28]. However, little has been described about the information related to the variation of Cu in serum related to the pathophysiology of metabolic diseases itself. 

The results regarding the association of overweight and obesity status with serum Cu levels observed in our study are supported by previous evidence [29,30,31,32,33,34]. As an example, studies in children and adolescents have reported elevated serum Cu levels in obesity compared to normal weight groups [29,30,31]. In adults, it has been seen that individuals with obesity showed higher Cu levels in comparison to normal weight individuals [32]. As well, a positive correlation between serum Cu level and BMI has also been found [33]. These findings are consistent with a meta-analysis where it was reported that excess body mass is associated with elevated serum Cu levels [34].

To understand the relationship between overweight, obesity, and serum Cu levels, it is important to keep in mind that most of the Cu levels in serum are associated with Cu-proproteins [35], of which, semicarbazide-sensitive amine oxidase (SSAO) and ceruloplasmin (Cp) are highly related to the pathophysiology of obesity [36,37]. Although SSAO is a Cu-dependent amine oxidase expressed in adipose tissue and involved in the uptake and storage of glucose and fatty acids [38], its increase in serum has been reported in association with obesity and hyperlipidemia [36]. A recent study evaluated the SSAO activity and Cu concentration in serum and adipose tissue of individuals that were a normal weight and obese [33]. This study reported that SSAO activity positively correlates with BMI and that Cu concentration in adipose tissue and serum correlates directly with SSO activity [33], which could suggest that the increase in adiposity that obesity involves, implies an elevation of SSAO activity in adipose tissue and serum, and consequently an elevation of Cu concentration in serum. In the case of Cp, it is an acute-phase plasma protein secreted by the liver in response to immune cells and has previously been reported in association with obesity in adult and children populations [37,39]. Nevertheless, there is evidence that obesity increases the secretion of proinflammatory cytokines from excess adiposity, especially the concentration of interleukin-6 (IL-6), which elevates the synthesis and secretion of Cp secreted by the liver [40]. Additionally, in previous studies, it has been reported that gene expression of Cp in adipose tissue is most enriched in obesity compared to individuals without obesity [41]. The case of the association of hypertriglyceridemia with the high level of serum Cu may also be explained by the serum concentration of the Cu-proproteins SSAO and Cp since both have been reported in positive association fasting plasma triglycerides in adults with metabolic syndrome [42] and in non-insulin-dependent patients with type 2 diabetes [36]. With this evidence, it may be possible to relate elevated Cu levels as another consequence of the pathophysiology of obesity. 

Evidence of association between triglycerides and Cu concentration in serum or dietary Cu intake is scarce. However, our results are consistent with those reported in Chinese adult women where it was observed that the serum Cu level was positively associated with the serum triglyceride concentration [43]. Another study in children and adolescents from the U.S. reported that higher dietary Cu intake increases the risk of hypertriglyceridemia [44]. Until now, the biological mechanism to explain the relationship between serum Cu levels and hypertriglyceridemia is still unclear. Nevertheless, previous studies have suggested that the higher levels of Cu may act as pro-oxidants that affect proper lipid metabolism. Excessive Cu concentration has been observed in association with excessive Cu chelate, which is directly related to inhibiting GSH reductase, reducing GSH levels, and impaired lipid metabolism [45,46].

Although high serum Cu level is widely associated with type 2 diabetes and metabolic syndrome [47,48,49], there is little evidence related to the association with IR. However, previous studies have shown a positive association between serum Cu levels and IR in children and adult populations [50,51]. Furthermore, in experimental animals with diabetes, has been seen that treatment with Cu chelators improves the metabolic condition and avoids metabolic complications related to this disease [52]. A possible mechanism that explains the association between IR and high serum Cu levels may be linked to the pro-oxidant effect of Cu. In an animal model, the serum Cu level has been reported in positive correlation with total reactive oxygen species level [52], which are widely related to the synthesis of pro-inflammatory cytokines and release of free fatty acids (FFA) [53], lending to IR and the risk to develop cardiovascular diseases [54]. Elevated FFA concentrations in circulation are related to increased diacylglycerols which activate signaling pathways that block insulin receptor substrate (IRS) phosphorylation, inhibiting insulin signaling and action [55]. Moreover, FFA can bind to Toll-like receptors 4 (TLR-4) in immune cells during obesity and activate the canonical nuclear factor kappa B (NF-κB) pathway, promoting the synthesis of proinflammatory cytokines that activate c-Jun N-terminal kinase (JNK) and suppressor of cytokine signaling (SOCS) impeding the binding of IRS 1/2 to the insulin receptor and disrupting insulin signaling [56,57]. FFA can also activate NADPH oxidase (NOX), triggering the formation of reactive oxygen species, followed by the production of pro-inflammatory cytokines that collectively promote an environment of inflammation and oxidative stress that induces IR [58]. 

Our study is one of the first reports of the association of overweight and obesity status, hypertriglyceridemia, and IR with serum Cu levels. However, these results are the product of an observational design. For this reason, we think it will be important to further evaluate the physiological mechanisms involved in these associations. Within the limitations of our study, we can mention that we analyzed individuals with overweight and obesity as a single group. Moreover, due to the high variability of the cut-off values to determine RI by TyG index, in the literature, it is still suggested to conduct more studies in populations with different ethnicities to consider the TyG index as a predictor of cardiovascular disease risk in particular populations. However, the TyG index has been used in different studies as a prognostic marker of cardiovascular disease. As an example, the TyG index has been reported in association with anthropometric markers related to overweight and obesity status (visceral fat and BMI) [59] and hypertriglyceridemia [60]. Regarding IR, the TyG index is widely used as a methodology for determining it. Such is the case in which the highest values of this index have been reported in association with harmful long-term cardiovascular events and estimated mortality risk from these causes, in important follow-up studies (123-month median follow-up) of large cohorts (16,613 individuals) [20]. Additionally, we are aware of the lack of homogeneity of our study sample (university students, professors, and potters). Studying different populations could represent different socioeconomic status that could influence the observed associations. Nevertheless, to avoid the possible effect of the socioeconomic status represented by the type of population, all association analyses in the study were adjusted by location, which is the same as the type of population. Another important limitation of our study is the lack of data related to dietary Cu intake. Previous studies describe that dietary Cu intake is directly related to serum Cu concentration. In this way, it will be interesting to evaluate in future studies whether the association of overweight and obesity status, hypertriglyceridemia, and IR with high serum Cu levels are replicated with dietary Cu intake in the Mexican population. Finally, the importance of our findings and future studies could help to understand the molecular mechanisms and treatment of metabolic diseases such as overweight and obesity status and their association with Cu. It would be important to perform follow-up studies with larger cohorts that can give us more clarity on the implications of Cu on obesity, IR, and cardiometabolic traits. 

## 5. Conclusions

Our results suggest a positive association of overweight and obesity status, hypertriglyceridemia, and IR with high serum Cu levels (≥129.397 µg dL^−1^) in the sample of Mexican adults studied. This observational study contributes to the knowledge related to the understanding of the factors associated with the pathophysiology of metabolic diseases in the Mexican population. However, it is important to perform further epidemiological and molecular studies to confirm the effects of Cu on metabolic diseases and understand its role as one of the possible markers of IR pathophysiology that could be intervened to improve insulin signaling.

## Figures and Tables

**Table 1:** General characteristics of study participants according to body weight status.

Trait	Normal Weight *n* = 107	Overweight and Obesity *n* = 239	*p*-Value
Women, *n* (%)	68 (63.6)	146 (61.1)	0.663
Age, years	37.22 ± 17.17	39.29 ± 15.27	0.264
BMI, kg/m^2^	22.834 ± 1.585	30.127 ± 4.953	**<0.001**
FPG, mg/dL	90.694 ± 9.215	94.676 ± 10.390	**0.001**
TC, mg/dL	165.220 ± 49.408	181.180 ± 51.520	**0.007**
TG, mg/dL	123.454 ± 54.515	158.746 ± 73.613	**<0.001**
TyG index	8.532 ± 0.475	8.838 ± 0.495	**<0.001**
Cu, μg dL^−1^	85.134 ± 69.906	116.171 ± 85.393	**0.001**

Variables with continuous data are expressed as mean ± standard deviation. The frequency of women is expressed as numerical (*n*) and percentage (%) frequency. Abbreviations: BMI, body mass index; FPG, fasting plasma glucose; TC, total cholesterol; TG, triglycerides; TyG, triglycerides glucose index. Student *t*- and Chi-square tests were used to compare means and frequencies, respectively. Significant *p*-values (*p*<0.05) are represented in bold.

**Table 2:** Association between serum Cu level and cardiometabolic traits.

Trait	Adjusted	
	Model 1	Model 2
BMI, kg/m^2^	0.001 ± 0.004 (0.745)	-
FPG, mg/dL	0.011 ± 0.006 (0.055)	0.008 ± 0.006 (0.175)
TC, mg/dL	0.030 ± 0.034 (0.388)	0.016 ± 0.035 (0.650)
TG, mg/dL	0.188 ± 0.045 **(<0.001)**	0.160 ± 0.045 **(<0.001)**
TyG index	0.002 ± 0.001 **(<0.001)**	0.001 ± 0.001 **(<0.001)**

Data are expressed as β value ± standard deviation. Abbreviations: BMI, body mass index; FPG, fasting plasma glucose; TC, total cholesterol; TG, triglycerides; TyG, triglycerides glucose index. Analysis by linear regression. Model 1: adjusted for age, sex, and locality; Model 2: adjusted for age, sex, locality, and overweight and obesity status. Significant *p*-values (<0.05) are represented in bold.

## Data Availability

The dataset generated during and/or analyzed during the current study are available from the corresponding author on reasonable request due to privacy.

## Appendix A

**Table A1:** Normality evaluation.

Trait	Shapiro–Wilk Test (*p*-Value)	
	Untransformed	Rank-Based Inverse Normal Transformation
BMI (kg/m^2^)	**<0.001**	0.999
FPG (mg/dL)	**0.01**	0.941
TC (mg/dL)	**<0.001**	0.992
TG (mg/dL)	**<0.001**	0.994
TyG index	**<0.001**	
Cu (µg dL^−1^)	**<0.001**	0.999

Shapiro–Wilk tests were performed on metabolic outcomes before and after rank-based inverse normal transformation to determine whether they were normally distributed. Significant *p*-values (<0.05) are represented in bold. Abbreviations: BMI, body mass index; FPG, fasting plasma glucose; TC, total cholesterol; TG, triglycerides; TyG index, triglycerides glucose index; Cu, copper.
